# Clinical applications and perspectives of circulating tumor DNA in gastric cancer

**DOI:** 10.1186/s12935-024-03209-4

**Published:** 2024-01-06

**Authors:** Jing-Han Li, Dan-Ying Zhang, Ji-Min Zhu, Ling Dong

**Affiliations:** Department of Gastroenterology and Hepatology and Shanghai Institute of Liver Diseases, Zhongshan Hospital, Fudan University, Shanghai, 200032 China

**Keywords:** Circulating tumor DNA, Liquid biopsy, Biomarker, Gastric cancer

## Abstract

Gastric cancer remains a leading cause of cancer-related death worldwide, largely due to inadequate screening methods, late diagnosis, and limited treatment options. Liquid biopsy has emerged as a promising non-invasive approach for cancer screening and prognosis by detecting circulating tumor components like circulating tumor DNA (ctDNA) in the blood. Numerous gastric cancer-specific ctDNA biomarkers have now been identified. CtDNA analysis provides insight into genetic and epigenetic alterations in tumors, holding promise for predicting treatment response and prognosis in gastric cancer patients. This review summarizes current research on ctDNA biology and detection technologies, while highlighting clinical applications of ctDNA for gastric cancer diagnosis, prognosis, and guiding treatment decisions. Current challenges and future perspectives for ctDNA analysis are also discussed.

## Introduction

Gastric cancer (GC) represents the fifth most common tumor and the fourth leading cause of cancer-related deaths worldwide [1]. According to World Health Organization statistics, the global incidence of GC is increasing continuously, from 1.09 million in 2020 to 1.77 million by 2040 [2]. As early GC is restricted to the mucosa and submucosa, the 5-year survival rate is over 90%. However, the prognosis is poor for advanced GC, with an average survival of only 12 months [3].

The diagnosis of GC is often made at an advanced stage due to the absence of early distinguishable symptoms and the need for a practical mass screening approach for the general population. Although serological tests, including pepsinogen I, pepsinogen II, pepsinogen ratio, gastrin-17, helicobacter pylori antibody, and carbohydrate antigen72-4 (CA72-4) [4], are less invasive, their sensitivity and specificity are limited. The Japanese GC Association has concluded that serum biomarkers are not helpful for early GC diagnosis but can be used to detect recurrence and distant metastases and to predict patient survival and postoperative recurrence [5]. Currently, the mainstay to confirm GC is endoscopy and tissue biopsy, both of which are invasive operations and dependent on the operator's skill. Thus, they are impractical for a mass screening program [6, 7]. Therefore, there is an urgent need for a less invasive, more sensitive, specific, and highly cost-effective test to improve the clinical utility for diagnosis, prognostic assessment, monitoring changes, and guiding treatment options.

**Fig. 1:**
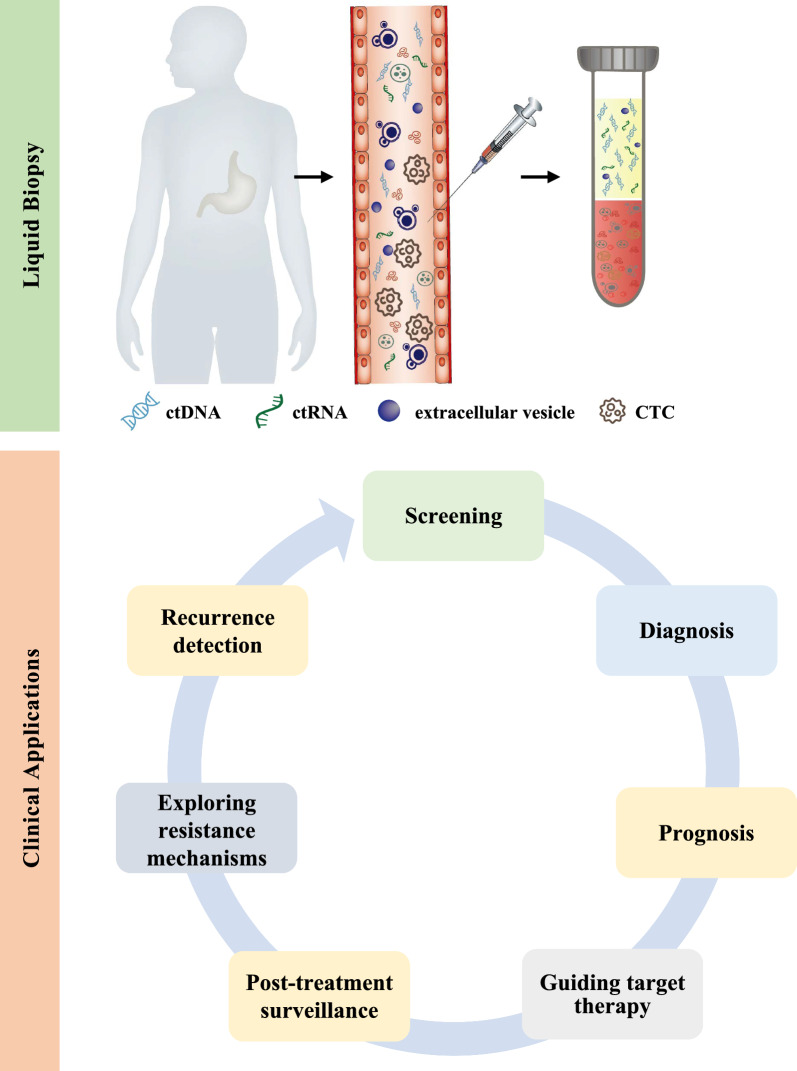
Clinical applications of liquid biopsy in gastric cancer. Liquid biopsy, including circulating tumor DNA (ctDNA), circulating tumor RNA (ctRNA), extracellular vesicle, and circulating tumor cell (CTC), has gained popularity as a valuable tool in clinical applications of gastric cancer

During the past decade, liquid biopsy has become a valuable tool in cancer detection by analyzing tumor-derived entities circulating in body fluids, determining the tissue of origin, monitoring prognosis, and assessing response and resistance to the treatment [8, 9]. These biomarkers include cell-free DNA (cfDNA), cell-free RNA, proteins, autoantibodies, circulating tumor cells, circulating tumor DNA (ctDNA), and cancer-derived extracellular vesicles [10]. Among them, ctDNA is the cornerstone of liquid biopsy in cancer applications due to its intimate relationship with tumors and has become a popular research topic in recent years [11, 12]. In this review, following a brief overview of the biology and detection technologies, we summarized the clinical applications of ctDNA, focusing on its potential in the diagnosis, prognosis, and therapy of GC (Fig. 1).

## Circulating tumor DNA

### The biological basis of ctDNA

cfDNA, identified by Mandel and Métais in 1948 [13], refers to extracellular DNA found in blood or body fluids, which can be either single-stranded or double-stranded [14]. In healthy individuals, cfDNA is primarily derived from apoptotic or necrotic cells or secreted from lymphocytes and other nucleated cells, which form small homogeneous DNA fragments less than 180 bp in length and 3.6–5.0 ng/mL in concentration. cfDNA has an estimated half-life between 16 min and 2.5 h, depending on factors such as the type and stage of the tumor [15].

In 1977, Leon et al*.* reported increased cfDNA derived from tumors [16]. After that, Stroun et al*.* demonstrated that cfDNA contained tumor-related mutations [17]. Therefore, cfDNA derived from tumors is described as ctDNA produced by lysed tumor cells or micrometastatic sites [18]. As a matter of principle, ctDNA contains the same genetic features as the tumor cells, such as single nucleotide mutations and methylation changes [19]. This distinguishes ctDNA from cfDNA and guides the development of cancer detection technologies. Since then, many studies have investigated the potential clinical utility of ctDNA analysis for various cancers [11, 12, 20, 21]. Researchers have gradually realized that the development of ctDNA research holds promise for advances in oncology diagnosis and prognosis prediction.

### Advantages and disadvantages of ctDNA testing

Tissue biopsy is currently considered the gold standard for diagnosing and treating cancers. It enables tumor classification, aggressiveness and progression assessment, and genetic composition and mutational phenotype analysis, thereby facilitating personalized treatment strategies [22]. However, ctDNA detection has several advantages over tissue biopsy. Firstly, tissue biopsy is invasive, expensive, and risks complications such as bleeding, local infection, and damage to adjacent tissues [23]. Sometimes, tissue biopsy is not feasible due to anatomic location or underlying coagulation dysfunction. There may also be an increased chance of false negative results due to the limited retrieval of the tumor tissue [24]. In contrast, ctDNA testing requires only a minimum of invasiveness to acquire cancer-related information, regardless of the location of the tumor. Secondly, tissue biopsy only provides information at a specific site and time point. At the same time, blood can be conveniently drawn for ctDNA testing at any time throughout the disease, thus allowing for real-time monitoring of tumor changes without the need for multiple invasive tissue biopsies or imaging surveillance. The short half-life of ctDNA makes it convincing for dynamic monitoring of disease progression [25]. Finally, analysis of ctDNA provides a comprehensive molecular profile of a patient's malignancy, thereby overcoming the challenges posed by intra-tumor heterogeneity and providing additional supportive information in the diagnosis and treatment selection [26].

Despite its potential, ctDNA has several drawbacks that impede its use. Firstly, ctDNA is generally present in low abundance in early-stage cancer and represents only a tiny fraction of total cfDNA (ranging from less than 0.1% to more than 10%), which is further diluted by DNA from non-tumor sources. Currently, detecting tumor-specific mutations on cfDNA is the only way of identifying the ctDNA [19]. Secondly, the proportion of ctDNA in total cfDNA depends on tumor load, cancer stage, cell renewal, and therapy response. It is estimated that patients with a tumor load of 100 g (about 3 × 10^10^ tumor cells) release 3.3% of their tumor DNA into circulation each day [27]. Hence, ctDNA is frequently undetectable in patients with a low tumor burden or at early stages. Thirdly, ctDNA fragments have a half-life of less than 2 h, requiring rapid processing and stringent pre-analytical procedures such as blood collection, transport, processing, and storage temperatures [28]. Fourthly, there is no consensus on standard experimental procedures for ctDNA assays, including sampling, storage conditions, cfDNA isolation and concentration, data analysis, and interpretation [29], leading to a lack of comparability between studies [30]. Finally, most current clinical studies are retrospective and small in sample size, highlighting an urgent need for multicenter, long-term prospective clinical trials to validate the feasibility of ctDNA in cancer detection, monitoring, and treatment [31].

### ctDNA detection methods

**Fig. 2:**
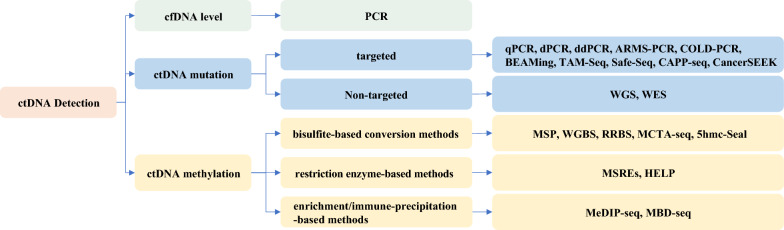
Detection methods of ctDNA in gastric cancer. Quantitative and qualitative changes of ctDNA in plasma provide valuable information for cancer. Quantitative change refers to the total ctDNA level, while qualitative changes include ctDNA mutations and methylation changes

Changes in ctDNA in plasma can be detected by quantitative and qualitative (Fig. 2). The former refers to total ctDNA concentration, while the latter refers to DNA aberrations such as single nucleotide mutations and methylation changes [32].

The qualitative analysis of ctDNA can be categorized into two types: targeted and non-targeted [33]. The former is restricted to the detection of single or several biomarkers, focusing on known genetic alterations in primary tumors, such as KRAS (Kirsten rat sarcoma viral oncogene), BRAF (v-Raf murine sarcoma viral oncogene homolog B1), and EGFR (epidermal growth factor receptor) [26]. On the other hand, the non-targeted analysis aims to screen the genome and identifies novel genomic abnormalities, usually through whole genome sequencing (WGS) testing. However, sensitive testing of large target regions is costly, so achieving an appropriate balance between target region size and test sensitivity is essential.

Initially, detecting specific mutations in ctDNA relied on standard quantitative reverse transcription polymerase chain reaction (qPCR). However, due to its limited sensitivity, qPCR was performed mainly in advanced patients with high ctDNA levels [34]. In cases of lower tumor load, where the percentage of ctDNA is significantly lower than 0.1%, digital PCR (dPCR) and droplet-based digital PCR (ddPCR) methods overcome these limitations. For example, Pearson et al*.* developed a screening tool based on recombinant fibroblast growth factor receptor 2 (FGFR2) ctDNA using ddPCR [35]. In addition, further high-resolution PCR-based methods that have been successfully applied to ctDNA analysis include the BEAMing (beads, emulsion, amplification, and magnetics) [36], ARMS-PCR (Amplification Refractory Mutation System PCR) [37], and COLD-PCR (co-amplification at lower denaturation temperature- PCR) at lower denaturation temperatures [38]. PCR-based technology is faster, less expensive, and highly sensitive, allowing for the detection of tumor-associated mutations at frequencies as low as 0.01% [26]. However, its main drawback is that a single test can detect only one or a few mutations, limiting its ability to study significant numbers and different kinds of genomic alterations [39]. In 2018, Cohen et al*.* developed a PCR-based test, CancerSEEK, and investigated its utility for the early detection of eight common cancers. The results showed that it could be used to assess cancer-specific characteristics in the early stages (I-III) of more than 82% of cancers [40].

Compared to PCR-based methods, next-generation sequencing (NGS)-based technology is characterized by high throughput, high sensitivity, and extensive coverage. It can identify somatic and germline mutations, copy number alterations, and other chromosomal rearrangements, including translocation, conversion, and inversion. Unlike targeted analysis, NGS does not require prior knowledge of the exact genetic changes in tumors, making it a non-targeted approach. Currently, targeted deep sequencing methods include TAM-Seq (tagged-amplicon deep sequencing) [41], Safe-SeqS (Safe-Sequencing) [42], and CAPP-Seq (Computer Aided Process Planning sequencing) [43]. These technologies allow NGS to provide personalized cancer genetic profiles and facilitate personalized medicine [19]. Based on this, Kato et al*.* demonstrated the feasibility of NGS for ctDNA evaluation in patients with gastroesophageal adenocarcinoma [44].

Whole exome sequencing (WES) and WGS can detect tumor mutations in all patients, making them ideal for genome-wide copy number analysis and detection of significant structural variants. However, their high cost renders them unsuitable for sensitively detecting single nucleotide variants [26]. Despite lower analytical sensitivity for ctDNA analysis throughout the disease course, WES and WGS can track clonal genomic evolution associated with tumor progression [45]. Li et al*.* developed fingerprinting profiles based on WES for ctDNA in individual patients. This study demonstrated that ctDNA fingerprinting improves the specificity of several tumor types for monitoring treatment response and sensitivity [46].

Although tumor-associated gene mutations have been the focus of biomarker research for a long time, their wide diversity has always been a challenge for developing validated biomarkers. To achieve sufficient sensitivity, a significant proportion of genomes must be examined [47]. In contrast, epigenetic alterations appear more stable and homogeneous in cancer, making them a promising alternative for biomarker development [48]. DNA methylation is the most widely studied epigenetic modification [49, 50]. There are two main types of methods for detecting ctDNA methylation, namely bisulfite-based conversion methods and non-bisulfite–based conversion methods. The latter includes restriction enzyme-based methods such as methylation-sensitive restriction enzymes (MSREs) [51], enrichment/immune-precipitation-based techniques such as methylated DNA immunoprecipitation sequencing (MeDIP-seq) [52], and 5-hydroxymethylation profiling. Many methylation detection methods based on bisulfite conversion have been developed, such as whole genome bisulfite sequencing (WGBS), reduced-representation bisulfite sequencing (RRBS), methylated CpG tandems amplification and sequencing (MCTA-seq), and methylation arrays [53].

## Clinical applications

### Diagnosis and screening

In the last few years, we have witnessed a growing body of clinical evidence supporting the detection of cfDNA for screening and monitoring patients with GC (Table 1). This test would be detected four years earlier than the current “gold standard” [54]. Plasma cfDNA levels in cancer patients, including GC patients, are two to three times higher than in healthy individuals [55]. However, plasma cfDNA levels may also increase in response to infection, inflammation, and other stressful conditions [56]. Therefore, quantifying plasma cfDNA would not be a sufficient biomarker to detect cancer due to its lack of specificity.

**Table 1:** Ongoing studies using ctDNA for detecting gastric cancer

NCT Number	Study type	Study title	Cohorts/Arms	Primary outcome measures	Numbers of enrollment
NCT04000425	OBSERVATIONAL	Potential Clinical Utilities of Circulating Tumor DNA in Gastric Cancer	Stomach adenocarcinoma patients who plan to receive radical gastrectomy	Disease recurrence risk; DFS; ctDNA changing to adjuvant chemotherapy response; Time of first negative ctDNA detection from positive ctDNA detection	55
NCT05029869	OBSERVATIONAL	Monitoring Minimal Residual Disease in Gastric Cancer by Liquid Biopsy Study Description	Patients with newly diagnosed and untreated gastric adenocarcinoma	The sensitivity and specificity of MRD detection using ctDNA as the biomarker	100
NCT04053725	OBSERVATIONAL	Prediction of the Efficacy of ctDNA in Immunotherapy for Advanced Gastric Cancer	Advanced Gastric Cancer Patients with second- or third-line chemotherapy and combined with immunoassay inhibitors	The proportions of patients with positive serum ctDNA that have postoperative relapse	200
NCT02887612	OBSERVATIONAL	ctDNA for Prediction of Relapse in Gastric Cancer	Early or intermediate-stage gastric cancer patients need to have surgical treatment	Positive Predictive Value; The proportions of patients with positive serum ctDNA that have postoperative relapse	200
NCT05208372	OBSERVATIONAL	Detection of CTC and ctDNA in the Diagnosis of Metastasis in Gastric Cancer	Borrmann III and Borrmann IV Gastric Cancer patients undergo either laparotomy or laparoscopic surgery	Quantity of CTCs; Expression of ctDNA	200
NCT04520295	OBSERVATIONAL	ctDNA Screening in Advanced HER2 Positive Gastric Cancer	HER2 positive gastric cancer patients	Change from baseline in molecular biomarkers (gene mutation, amplification and fusion) at time on best overall response	100
NCT04943406	OBSERVATIONAL	Peritoneal Lavage Liquid Biopsy in Patients with Gastric Cancer	Patients with histologically proven gastric or GEJ Siewert type II and III adenocarcinoma	Prognostic impact (overall survival and disease-free survival) of ctDNA positivity	150
NCT03425058	OBSERVATIONAL	Molecular Evaluation of Neoadjuvant Chemotherapy for Locally Advanced Gastric Cancer	Locally advanced gastric cancer patients without distant metastasis or peritoneal dissemination (T4a/T4bN + M0)	The relationship between dMMR/MSI status and response to neoadjuvant chemotherapy; The concordance and accuracy of response evaluation results determined by ctDNA, CTCs compared with imaging and serum tumor biomarkers (CEA, CA19-9, CA72-4 et al.)	80
NCT05661110	OBSERVATIONAL	Biomarker Analysis of HIPEC Combined with PD1/PDL1 Inhibitor for Gastric Cancer with Peritoneal Metastasis	Advanced gastric adenocarcinoma confirmed by histology with peritoneal metastasis	Numerical changes and genomic changes of ctDNA during treatment	46
NCT04511559	OBSERVATIONAL	Methylation Analysis of Circulating Tumor DNA in Gastric Cancer	Healthy volunteers and Gastric Cancer patients with upper GI endoscopy for standard clinical indications	Analysis ctDNA methylation status and its Correlation to early diagnosis and prognostic evaluation of gastric cancer	540
NCT05431621	OBSERVATIONAL	Establishment of Molecular Classification Models for Early Diagnosis of Digestive System Cancers	Newly-diagnosed patients with digestive system cancers	Establish ctDNA-targeted sequencing models for early detection of esophageal, gastric, colorectal and hepatocellular cancer, and evaluate the diagnosis value	2430
NCT05027347	OBSERVATIONAL	Detection of Plasma Circulating Tumor DNA in Gastric Cancer	Patients diagnosed with early and locally advanced stage (I, II and IIIA) gastric cancer	The sensitivity and and specificity of our mutation-based assay for detecting early-stage gastric cancer patients	200
NCT05227261	OBSERVATIONAL	Early Detection of Five Common Cancers Using the ctDNA Analysing Test	Patients with no history of cancer	Positive predictive value, Negative predictive value, sensitivity and specificity of the blood ctDNA test in early detecting cancers	3000
NCT04947995	OBSERVATIONAL	Multi-Omics Noninvasive Inspection of Tumor Risk for Gastric Cancer	Subjects aged ≥ 40 who will receive EGD	The sensitivity and specificity of blood-based multi-omics assay for early detection of gastric cancer	450
NCT05224596	OBSERVATIONAL	AssesSment of Early-deteCtion iqui oN iquid Biopsy in GASTRIC Cancer, ASCEND-Gastric	participants in cancer with new diagnosis of gastric cancer; participants in benign disease with new diagnosis of benign gastric disease	Sensitivity and specificity of the cfDNA methylation-based model in detecting gastric cancer	498
NCT05513144	OBSERVATIONAL	Potential Clinical Utilities of Circulating Tumor DNA in Advanced HER2 Negative Gastric Cancer	HER2 negative gastric cancer patients	Prognostic molecular markers; The sensitivity and specificity of ctDNA detection	30
NCT02838836	OBSERVATIONAL	Tumor Cell and DNA Detection in the Blood, Urine and Bone Marrow of Patients with Solid Cancers	Subjects with the diagnosis of a solid cancer of all stages will be included (lung, esophageal, stomach, bile duct/pancreas, colorectal, melanoma, sarcoma)	CTC/DTC numbers measured in blood, urine and bone marrow samples correlated with patient outcome	120
NCT04576858	OBSERVATIONAL	Clinical Utility of Circulating Tumor DNA in Gastro-Esophageal Cancer	Patients with gastroesophageal cancer	Time to recurrence	1950
NCT04385316	OBSERVATIONAL	Clinical Study of Gastric Cancer, Colorectal Cancer and Bladder Cancer Based on Liquid Biopsy	Gastric cancer, colorectal cancer or bladder cancer patients in Jiangsu Provincial People’s Hospital	Tumor related mutation spectrum in different patients	3
NCT03517332	OBSERVATIONAL	Circulating Tumor DNA Exposure in Peripheral Blood	Subjects that are diagnosed with a malignancy (cohort 1); Subjects that have not been diagnosed with a malignancy (cohort 2)	test the feasibility of a novel process for the detection of circulating tumor DNA	10,000
NCT02674373	OBSERVATIONAL	Prognostic and Predictive Impact of Circulating Tumor DNA in Gastric Cancer Treatment	patients treated for a histologically proven, localized or advanced adenocarcinoma of gastric or gastro-oesophageal junction	PFS	200
NCT05482516	INTERVENTIONAL	Evaluating Novel Therapies in ctDNA Positive GI Cancers	Patients with GI Cancers	Rates of SignateraTM ctDNA positive Patient identification; Rate of ctDNA Complete Response (CR); Rate of ctDNA Partial Response (PR); Rate of ctDNA Progression of Disease (POD) or Clinical/radiographic Relapse	20
NCT03957564	INTERVENTIONAL	Liquid Biopsy in Monitoring the Neoadjuvant Chemotherapy and Operation in Gastric Cancer	Patients with resectable or locally advanced gastric or gastro-oesophageal junction cancer (> T1 and N +) without distant metastases (M0)	Numbers and types of CTC pre- and post- neoadjuvant chemotherapy and after operation; Mutation rate and concentration of ctDNA pre- and post- neoadjuvant chemotherapy and after operation	40
NCT04929015	INTERVENTIONAL	Peritoneal Carcinomatosis Leveraging ctDNA Guided Treatment in GI Cancer Study (PERICLES Study)	Patients with GI Cancers with documented diffuse peritoneal carcinomatosis	Clearance rate of ctDNA with cytoreductive surgery (CRS), comparing with clinical staging of CRS	30
NCT04510285	INTERVENTIONAL	A Single-Arm Pilot Study of Adjuvant Pembrolizumab Plus Trastuzumab in HER2 + Esophagogastric Tumors with Persistent Circulating Tumor DNA Following Curative Resection	ECOG performance status 0–2	Rate of ctDNA Clearance at 6 months with treatment	1
NCT05594381	INTERVENTIONAL	A Biomarker Study for Predicting the Efficacy of Neoadjuvant Sintilimab Plus SOX for Gastric Adenocarcinoma	G/GEJ adenocarcinoma patients with cStage III	Pathological complete response rate (pCR)	90
NCT05494060	INTERVENTIONAL	XELOX Combined with Anlotinib and Penpulimab vs XELOX as Adjuvant Therapy in ctDNA Positive Gastric and Esophagogastric Junction Adenocarcinoma	ECOG performance status score 0–1; Histologically or cytologically confirmed GC or GEJ carcinoma, had been treated with Radical resection (D2, R0 or R1); Pathological stage:II-III (8th AJCC TNM)	DFS	80
NCT05348161	INTERVENTIONAL	Dynamic Multiomics Evaluation of Anti-HER2 and Immunotherapy in HER2 Positive Gastric Cancer	Patients histologically confirmed of unresectable recurrent or metastatic gastric adenocarcinoma with HER2 overexpression confirmed by IHC or ISH	Proportions and numbers of HER2 & PD-L1 positive CTC; Incidence rate of ctDNA deletion, amplification, insertion and other types of variation evaluated	100
NCT04162665	INTERVENTIONAL	MR-guided Pre-operative RT in Gastric Cancer	Newly diagnosed histologically or cytologically gastric adenocarcinoma	Complete pathologic response (pCR—primary and nodal) rate	36

Information on tumor-associated genetic variants can be detected in ctDNA, ranging from simple point mutations to complex structural variants and even chromosomal copy number variants [57]. Therefore, detecting tumor-associated mutations in ctDNA can provide more identification of GC and guide its detection. Bettegowda et al*.* [58] first caught ctDNA containing tumor-specific single nucleotide variants in the plasma of 15 GC patients. Following this, Fang et al*.* [59] analyzed eight genetic alterations and found that Tumor Protein 53 (TP53), AT-Rich Interaction Domain 1A (ARID1A), and phosphatidylinositol-3-kinase catalytic subunit α (PI3KCA) were the most frequently mutated in ctDNA of patients with advanced GC. The detection rate of ctDNA was found to correlate with the tumor stage. Tumor-specific TP53 mutations were detected in patients with stage III-IV GC but not in patients with stage II GC [60]. It has been demonstrated that the copy number of Human epidermal growth factor receptor-2 (HER2) in the plasma of GC patients is significantly higher than that of healthy controls [61]. However, Kinugasa et al*.* [62] found low concordance between HER2 levels in tumor tissue and plasma DNA. This discordance may be caused by intra-tumor heterogeneity or sampling error due to low ctDNA levels [26]. To investigate whether ctDNA can cover tumor heterogeneity, Gao et al*.* [63] performed paired sequencing of tumor tissue biopsies and plasma samples from five patients. The biopsies confirmed the presence of tumor heterogeneity, but ctDNA only partially covered this heterogeneity. These analyses suggest that ctDNA research may be superior to tissue biopsy when examining GC with extensive intra-tumor heterogeneity.

In addition to single nucleotide variants, many studies have evaluated ctDNA methylation as a potential biomarker for cancer detection. It has been suggested that epigenetic alterations often precede somatic mutations and are more common than previously thought [64]. Circulating cfDNA methylation is highly predictive for GC, compared to methylation biomarkers in tissues [65]. Hypermethylation of p16 and E-calmodulin gene promoter regions has been detected in serum DNA samples from GC patients but not in healthy volunteers [66]. However, the reported ratio of ctDNA p16 promoter methylation in GC varies significantly across different studies [67], indicating the need for further validation. Ras association domain family 1, form A (RASSF1A), and protocadherin 10 (PCDH10) are tumor suppressor genes. Hypermethylation of RASS1A and PCDH10 was detectable in plasma samples from GC patients [68]. The study by Bernal et al. [69] confirmed the high frequency of methylation of seven genes in GC plasma, including Adenomatous Polyposis Coli (APC), SH2 domain-containing protein tyrosine phosphatase 1 (SHP1), E-calmodulin, Estrogen receptor (ER), Reprimo, Semaphorin-3B (SEMA3B) and 3-O-sulfotransferase-2 (3OST2). Additionally, methylation of tissue factor pathway inhibitor 2 (TFPI2) [70], XIAP associated factor 1 (XAF1) [71], Reprimo-like (RPRML) [72], multiple tumor suppressor 1 (MTS) and Cadherin 1 (CDH1) promoter region [10] dedicator of cytokinesis 10 (DOCK10), calcineurin binding protein 1 (CABIN1) and KQT-like subfamily, member 5 (KCNQ5) [73] can all be used as potential non-invasive diagnostic indicators in GC. In a meta-analysis of 16 studies, Gao et al*.* [74] demonstrated a significant association between ctDNA methylation levels and various parameters with high specificity and relatively moderate sensitivity, such as TNM (Tumor Node Metastasis) stage, tumor load, lymph node metastasis, and distant metastasis in GC patients. Runt-related transcription factor 3 (RUNX3) methylation in ctDNA is a valuable biomarker for detecting early GC [75]. The RUNX3 methylation [76] and secreted frizzled-related protein 2 (SFRP2) methylation [77] index coordinates with cancer stage, lymphatic and vascular invasion and is more sensitive than carbohydrate antigen (CEA) as a biomarker.

**Fig. 3:**
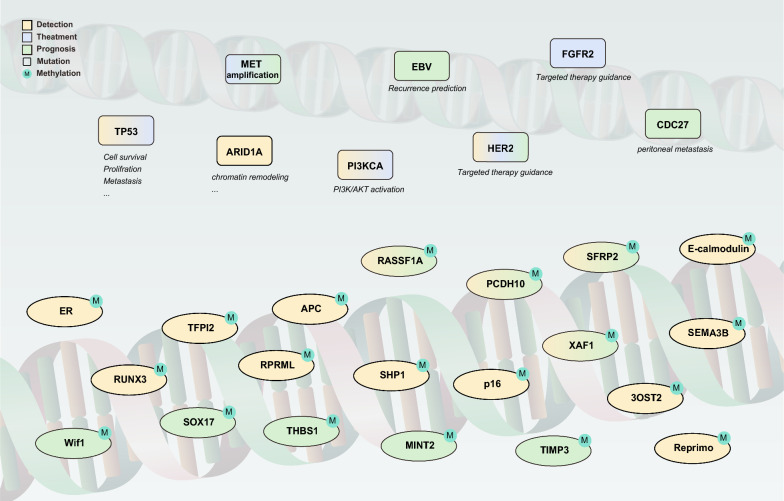
High-frequency genetic markers associated with gastric cancer and their involvement in key cellular pathways

It has been demonstrated that ctDNA methylation can detect GC early and track cancer progression. Lin et al*.* [78] measured the methylation status of three selected genes in blood samples from GC and precancerous patients using the methylation-specific PCR (MSP) assay. They found that the methylation rates of Zic family member 1 (ZIC1), homeobox D10 (HOXD10), and RUNX3 were significantly increased during gastric carcinogenesis. Combining these three genes showed a synergistic effect in identifying GC and precancerous lesions, compared to testing individual biomarkers [10]. The analysis of methylated ctDNA sites combined with the study of other cancer-related changes in DNA can also significantly improve cancer diagnosis [79]. Therefore, combinations of multiple methylation sites or combinations of methylation with other mutations provide a new idea to improve the test's specificity. Although many methylation sites associated with GC have already been identified, it is necessary to explore the differentially methylated sites between GC and normal groups further for screening and surveillance purposes (Fig. 3).

Therefore, changes in ctDNA levels can be used to detect GC, but more is needed as a biomarker for detecting GC due to their lack of specificity. Detection of tumor-associated mutations (e.g., TP53, HER2, ARID1A, and PI3KCA) may identify ctDNA more specifically and thus guide GC diagnosis. ctDNA methylation can also diagnose GC and assess tumor load. The specificity of GC detection is improved by using a combination of multiple methylation sites or combining ctDNA methylation sites with other mutations. ctDNA may be superior to conventional tissue biopsy because it overcomes false-negative detection due to intra-tumor heterogeneity of tissue biopsy.

### Evaluation of prognosis

Post-treatment surveillance aims to detect asymptomatic recurrence, early treatment, and improve survival. Current post-treatment surveillance and prognosis assessment methods are imaging and endoscopic biopsy, which have disadvantages such as radiation, invasiveness, and high-cost [80]. As an alternative, blood biomarkers can provide valuable prognostic information for GC. For example, the sensitivity of CEA, carbohydrate antigen 19-9 (CA19-9), and carbohydrate antigen 72-4 (CA72-4) ranges from 30.8–57.1% [81, 82]. Additionally, plasma-based ctDNA monitoring is more sensitive than conventional imaging for detecting recurrence, as ctDNA level, mutation status, and methylation levels can vary dynamically with the treatment [83].

A meta-analysis on the association of ctDNA and the prognosis of GC showed that detecting ctDNA could be a promising predictor in GC patients [84]. The changes in ctDNA levels are reliable in assessing the prognosis of GC. High ctDNA levels are associated with peritoneal recurrence and poor prognosis in advanced GC patients [59]. The ctDNA level decreased significantly 24 h after surgery [85] but increased again if the patient experienced tumor recurrence or progression [86]. A large study of 428 GC patients carried out by Lan et al*.* [87] found that persistently high ctDNA levels after resection were more sensitive than CEA in predicting recurrence. Postoperative ctDNA was significantly associated with recurrence up to 12 months after surgery. However, no correlation was found between preoperative ctDNA levels and recurrence. This suggested the clinical usefulness of postoperative ctDNA monitoring for cancer recurrence [88].

ctDNA levels were also associated with disease-free survival (DFS) in advanced GC patients 3 months after receiving systemic chemotherapy. Patients with lower ctDNA levels had significantly longer DFS [89]. Furthermore, changes in ctDNA levels after treatment can predict treatment response and progression-free survival (PFS), with lower levels of ctDNA being associated with improved outcomes [90]. ctDNA testing is capable of detecting "molecular recurrence" earlier than an imaging-based diagnosis in cases of post-treatment tumor recurrence [91], providing a potential therapeutic window to advance further treatment [92]. However, it has also been shown that ctDNA monitoring during chemotherapy and post-operation does not appear to be a valuable tool for predicting efficacy and recurrence, mainly due to the poor sensitivity of ctDNA testing [93]. Therefore, developing new methods to improve the sensitivity of ctDNA detection may be the direction of further exploration.

Changes in ctDNA profile are closely related to treatment outcome and disease progression recurrence, thereby serving for prognostic assessment. Early detection of recurrence during follow-up allows early intervention, leading to a better treatment efficacy [26]. Postoperative tumor-informed ctDNA detection in EGC is feasible and allows for enhanced patient risk stratification and prognostication during curative-intent therapy [94]. GC patients with high ctDNA mutation abundance exhibited shorter overall survival (OS) than those with low mutation abundance [95]. Reduced ctDNA mutation frequency after treatment was associated with improved PFS and OS [96]. Patients with peritoneal metastases have more ctDNA mutated genes than non-peritoneal metastases. Mutations in cell division cycle 27 (CDC27) are associated with a higher risk of peritoneal metastases and a lower survival rate [97]. Patients with Mesenchymal-epithelial transition (MET) amplification in ctDNA have shorter OS than those without MET amplification, which indicates that ctDNA can predict disease progression in patients with advanced GC [98]. In some patients with Epstein-Barr virus (EBV)-associated GC, circulating EBV DNA is reduced after surgery and increases before clinically detectable recurrence. This could help monitor tumor load in patients with EBV-associated GC and predict recurrence [99]. HER2 alterations in ctDNA were significantly associated with poor OS [44]. Patients who tested positive for HER2 ctDNA before treatment had significantly shorter survival than those with negative. Still, no difference in survival was found when comparing the survival of patients regardless of tissue HER2 status [62]. This may be due to tumor heterogeneity, but ctDNA testing may provide a more accurate assessment. Based on a special NGS panel, the number of ctDNA mutations before the start of first-line chemotherapy has prognostic value. Moreover, residual ctDNA after three cycles of systemic treatment is associated with an inferior survival [100]. Changes in genomic features of ctDNA could be biomarkers for predicting the response of platinum-based first-line chemotherapy in patients with advanced GC [101]. Although many changes in genomic features of ctDNA have already been identified, it is necessary to explore more genomic changes further.

The MSP assay was applied to assess the value of the early diagnosis of recurrent disease in patients with GC. Nearly half of the patients showed aberrant methylation in plasma samples [102]. The transition of negative XAF1 methylation to positive in postoperative serum was strongly associated with tumor recurrence [71]. Aberrant methylation of Munc18-1 interacting protein2 (MINT2) promoter [103] and BVES (THBS1) [104] in ctDNA was associated with the peritoneal spread and tumor progression, which could be considered as potential poor prognostic factors for GC patients. The cumulative survival rates of ctDNA RASSF1A methylation and ctDNA PCDH10 methylation-positive cases were significantly lower than those of negative cases [68]. However, some studies found no correlation between RASSF1A promoter methylation and clinical outcomes [105], thus necessitating further research to validate the relevant findings. In addition to RASSF1A, Sex determining region Y-box 17 (SOX-17) and WNT inhibitory factor 1 (Wif-1) methylation were also associated with a decrease in PFS and OS [106]. In stage III and IV GC patients, PFS and OS were shorter in those with hypermethylated SFRP2 [77]. Methylation of tissue inhibitor of metalloproteinase-1 (TIMP-3) was associated with poorer DFS [107]. Therefore, detecting ctDNA methylation may provide a new assessment strategy for GC prognosis.

In summary, changes in ctDNA levels after treatment can predict the prognosis of GC patients. Patients with ctDNA mutations in GC have a worse prognosis than those without or with lower ctDNA mutations. ctDNA methylation detection may also provide a new assessment strategy for GC prognosis.

### Treatment

#### Guiding target therapy

Current treatments for GC include surgery, chemotherapy, radiotherapy, and targeted therapies against vascular endothelial growth factor receptor (VEGFR, ramucirumab) and HER2 (trastuzumab) [108]. Genomic analysis of ctDNA can identify therapeutic targets. Combined with information on tumor load or aggressiveness in ctDNA, it is possible to predict the need for preoperative chemotherapy, surgery, and postoperative chemotherapy. Repeat analysis of ctDNA during treatment can also track changes in tumor genomic profiles [26]. Molecular heterogeneity is a significant challenge in biomarker-based clinical trials for cancer patients [109]. Still, ctDNA analysis can help to avoid false negative results caused by intra-tumor heterogeneity, especially in patients with metastatic GC.

In the context of metastatic GC, genomic analysis of ctDNA may be more suitable than primary tumor biopsy for identifying targetable aberrations, thus more accurately guiding the targeted cancer therapy [26]. Identification of genomic alterations, e.g., TP53, LDL receptor-related protein 1B (LRP1B), HER2, and KRAS mutations, blood tumor mutation burden, and blood microsatellite instability status can provide recommendations for the clinical decision of advanced GC [98]. Analysis of HER2 copy number changes in ctDNA enables real-time assessment of HER2 status, which can be used to monitor the efficacy of trastuzumab and guide treatment selection. This approach can overcome the challenge of heterogeneity and is more effective than commonly used CEA and CA19-9 [110, 111]. By detecting HER2 status during tumor progression and treatment, clinicians can make proper decisions regarding molecularly targeted therapy for GC patients [112].

The levels of PIK3CA mutation in ctDNA also correlated with drug response and disease progression better than CEA, emphasizing the utility of ctDNA in monitoring treatment response and disease progression [113]. ctDNA sequencing identified fibroblast growth factor receptor-2 (FGFR2) amplification, which is undetected by tissue testing in patients with advanced GC [114]. Patients with high levels of FGFR amplification in ctDNA responded to treatment such as the FGFR inhibitor AZD4547 [35].

#### Exploring resistance mechanisms

ctDNA can monitor treatment response and identify resistance mutations during chemotherapy. Early detection of treatment resistance may allow modification in therapy to improve patient prognosis or discontinue treatment to avoid adverse effects [26]. Longitudinal ctDNA sequencing provides new insights into genetic alterations of trastuzumab resistance in HER2-positive GC patients. By tracking changes in HER2 copy number, the main mechanisms of primary or acquired resistance can be distinguished [115]. ctDNA sequencing is performed during anti-HER2 therapy and identified 32 extended mutations that may be associated with trastuzumab resistance. Further studies targeting these mutations could improve treatment strategies for patients [116].

Changes in the number of mutations and copy number levels of the gene were associated with the treatment effect. A significant difference in the incidence of TP53 mutations was found between the ineffective and effective groups [95]. Mesenchymal to epithelial transition factor (MET) amplification occurs in approximately 5% of GC patients. A strong correlation between high MET copy number in ctDNA and the response to MET inhibitors, such as Voritinib [117], suggests using ctDNA to guide treatment decisions and assess prognosis in GC patients [118].

The analysis of ctDNA by NGS has revealed several mutations that lead to therapeutic resistance during disease progression. These include recurrence of MET amplification, multiple secondary MET mutations (including D1228, Y1230, V1092, G1163, and L1195), and significant increases in the relative copy number of the FGFR2 gene. These studies suggest that ctDNA analysis can provide quantitative information about the development of therapeutic resistance and can also be used to explore the resistance mechanisms [119, 120].

## Perspectives

Analysis of ctDNA has the potential to be applied in the detection, evaluation of prognosis, and therapeutic guidance of cancer (Table 2). A standardized ctDNA assay has yet to be identified, so cross-sectional comparisons between studies are currently unavailable. Methodological differences among studies, such as variations in blood collection tubes, storage time and temperature, DNA isolation methods, and the nature of the analysis (automated or manual), may affect the results of meta-analysis, leading to false positives or false negatives. For instance, a study comparing different blood collection tubes to analyze epigenetic alterations in ctDNA found that some could only be refrigerated for 24 h, while others could be stored at room temperature for 48 h [121]. Additionally, the use of plasma or serum may introduce differences in results, as serum may have a high DNA yield due to contamination of the sample with DNA from leukocytes [122]. Therefore, it is crucial to specify an optimal set of methods for ctDNA collection, storage conditions, extraction, and analysis to ensure comparability among studies and greater convenience in the clinical research [29].

**Table 2:** ctDNA as biomarkers in gastric cancer

Type	Gene	Expression	Function	Sample type	Case number	GC information	Sensitivity (%)	Specificity (%)	AUC	Method	References
Mutation	TP53	High	Detection	Plasma	277 GC	TNM: I: 18; II: 76; III: 150; IV: 33				qPCR	[59]
		High	Treatment	Plasma	63 GC	TNM: III: 20; IV: 43				NGS	[98]
		High	Treatment	Plasma	23 GC	TNM: T1: 3; T2: 8; T3: 18; T4: 3; N0: 15; N + : 7;				NGS	[95]
	ARID1A	High	Detection	Plasma	277 GC	TNM: I: 18; II: 76; III: 150; IV: 33				qPCR	[59]
	PI3KCA	High	Detection	Plasma	277 GC	TNM: I: 18; II: 76; III: 150; IV: 33				qPCR	[59]
		High	Treatment	Plasma	56 GC	Tumour location: EGJ: 21(37.5%); Non-EGJ: 35(62.5%); Lauren classification: Intestinal type: 37(66.1%); Diffuse type: 11(19.6%); Mixed type: 8(14.3%)				ddPCR	[113]
	HER2	High	Detection	Plasma	81 GC, 103 controls	Lauren classification: Intestinal: 35; Diffuse 42; Mixed: 4 Tumor stage: EGC: 36; AGC: 45	87.7	64.1	0.744	PCR	[61]
		High	Detection/prognosis	Serum	24 GC	TNM: III: 1; IV: 23				ddPCR	[62]
		High	Treatment	Plasma	63 GC	TNM: III: 20; IV: 43				NGS	[98]
		High	Treatment	Plasma	60 GC, 30 Healthy controls	pTNM: I/II: 10; III: 50	73.3	93.3		ddPCR	[110]
		High	Treatment	Plasma	52 GC, 40 Healthy controls	TNM: II: 10; III 40; IV: 2	69.2	80.0	0.803	rqPCR	[112]
		High	Treatment	Plasma	78 GC	TNM: I: 13; II: 16; III: 33; IV: 16 Lauren type: Diffuse: 12; Intestinal: 42; Mixed: 24				targeted sequencing	[115]
		High	Treatment	Plasma	21 AGC	TNM: IIIA: 2; IIIC: 2; IV: 17 Lauren type: Diffuse: 4; Intestinal: 7; Mixed: 10				targeted capture sequencing	[116]
	CDC27	High	Prognosis	Plasma	63 GC	TNM: I/II: 14; III: 49				WES	[97]
	MET amplification	High	Prognosis	Plasma	63 GC	TNM: III: 20; IV: 43				NGS	[98]
		High	Treatment	Plasma	1 GC	TNM: IV:1				NGS	[119]
		High	Treatment	Plasma	3 GC					NGS	[120]
	EBV	High	Prognosis	Plasma	153 GC	pTNM: I/II: 56; III: 97				PCR	[99]
	FGFR2	High	Treatment	Plasma	365 GC					NGS	[114]
		High	Treatment	Plasma	341 GC					ddPCR	[35]
Methylation	p16	Hyper	Detection	Serum	109 GC, 10 Healthy controls	TNM: I/II: 67; III/IV: 42	18	100	-	MSP	[66]
	E-calmodulin	Hyper	Detection	Serum	109 GC, 10Healthy controls	TNM: I/II: 67; III/IV: 42	24	100	-	MSP	[66]
		Hyper	Detection	Plasma	43 GC	EGC: 5 AGC:38				MSP	[69]
	RASSF1A	Hyper	Detection/prognosis	Plasma	101 GC, 202 Healthy controls	TNM: I/II/III: 23; IV: 54	83.2	94.55	-	MSP	[68]
			Prognosis	Plasma	70 GC	Lauren type: Diffuse: 23; Intestinal: 32; Mixed: 15				MSP	[106]
	PCDH10	Hyper	Detection/prognosis	Plasma	101 GC 202 Healthy controls	TNM: I/II/III: 23; IV: 54	94.1	97.03	-	MSP	[68]
	APC	Hyper	Detection	Plasma	43 GC	EGC: 5 AGC:38				MSP	[69]
	SHP1	Hyper	Detection	Plasma	43 GC	EGC: 5 AGC:38				MSP	[69]
	ER	Hyper	Detection	Plasma	43 GC	EGC: 5 AGC:38				MSP	[69]
	Reprimo	Hyper	Detection	Plasma	43 GC	EGC: 5 AGC:38				MSP	[69]
	SEMA3B	Hyper	Detection	Plasma	43 GC	EGC: 5 AGC:38				MSP	[69]
	3OST2	Hyper	Detection	Plasma	43 GC	EGC: 5 AGC:38				MSP	[69]
	TFPI2	Hyper	Detection	Serum	73 GC	TNM: I/II: 33; III/IV: 40				qMSP	[70]
	XAF1	Hyper	Detection/prognosis	Serum	202 GC, 88 Healthy controls	TNM: I/II: 64; III/IV: 138	69.8	100	0.91	MSP	[71]
	RPRML	Hyper	Detection	Plasma	90GC, 25 Healthy controls	TNM: I/ II: 32; III/IV: 58 Lauren type: Diffuse: 31; Intestinal: 28; Mixed: 13	56	88	0.726	MethyLight assay	[72]
	RUNX3	Hyper	Detection	Serum	202 GC, 850 Healthy controls	TNM: I: 21; II: 43; III: 117; IV: 21	70.8 95.5	99.8 62.5	0.854	rt‐MSP	[75]
		Hyper	Detection	Serum	65 GC, 30 Healthy controls	TNM: I: 28; II: 19; III: 14; IV: 13	95.5	62.9	0.8651	RTQ-MSP	[76]
	SFRP2	Hyper	Detection/prognosis	Plasma	148 GC	TNM: I: 4; II: 23; III: 63; IV: 58				PCR	[77]
	ZIC1 + HOXD10 + RUNX3	Hyper	Detection	Plasma	131 GC, 120 controls	TNM: I/II: 63; III/IV: 58	91.6	50		MSP	[78]
	MINT2	Hyper	Prognosis	Peritoneal lavage fluid/blood	92 GC	TNM: I/II: 46; III/IV: 46				rt‐MSP	[103]
	THBS1	Hyper	Prognosis	Peritoneal lavage fluid/serum	92 GC	TNM: I/II: 46; III/IV: 46				MSP	[104]
	SOX-17	Hyper	Prognosis	Plasma	70 GC	Lauren type: Diffuse: 23; Intestinal: 32; Mixed: 15				MSP	[106]
	Wif-1	Hyper	Prognosis	Plasma	70 GC	Lauren type: Diffuse: 23; Intestinal: 32; Mixed: 15				MSP	[106]
	TIMP-3	Hyper	Prognosis	Peritoneal lavage fluid/serum	92 GC	TNM: I/II: 46; III/IV: 46				rt-MSP	[107]

To detect precancerous lesions and early cancers, there will most likely not be enough ctDNA in the plasma due to low disease burden. The low concentration of ctDNA can be compensated by developing novel reagents and methods for ctDNA isolation and extraction to improve ctDNA capture efficiency and reduce costs. Combining analytes can achieve the sensitivity and specificity required for robust early detection assays. For example, the specificity of detection is improved by using ctDNA methylation site combinations or methylation in combination with other mutations or by using ctDNA in combination with other biomarkers such as CEA, CA19-9, and CA72-4.

Another major obstacle to using ctDNA testing as a screening method is our desire to identify multiple cancer types without prior knowledge of any particular cancer mutation. Given the high costs of ctDNA studies, assessing all coding regions in cancer-associated genes is unrealistic. While the cost of sequencing will decrease over time, current methods may reduce the cost of ctDNA testing by focusing on mutations or methylation of specific genes. As a result, current ctDNA testing methods are unlikely to detect uncommon cancers with unusual cancer characteristics [28].

Analysis of ctDNA has been shown to provide information on mutations that are not found in tissue biopsies due to intra-tumor heterogeneity, which can help stratify patients for testing targeted drugs and may also help identify new therapeutic targets. Moreover, most current clinical studies on ctDNA are retrospective, with small sample sizes. All these must be explored in more multicenter and long-term prospective clinical trials [26].

## Conclusions

GC remains one of the most common malignancies worldwide with a poor prognosis, primarily due to the lack of population-appropriate screening, early detection methods, and suitable treatment options. The application of ctDNA as a biomarker is an exciting and emerging area for disease screening and monitoring in GC. Moreover, combining ctDNA with other biomarkers is expected to enhance cancer management for GC patients in the near future.

## Data Availability

Not applicable.

## Abbreviations

CA: Carbohydrate antigen
CEA: Carcinoembryonic antigen
cfDNA: Cell-free DNA
ctDNA: Circulating tumor DNA
DFS: Disease-free survival
EBV: Epstein-Barr virus
FGFR2: Fibroblast growth factor receptor 2
GC: Gastric cancer
HER2: Human epidermal growth factor receptor-2
MET: Mesenchymal-epithelial transition
OS: Overall survival
PCDH10: Protocadherin 10
PCR: Polymerase chain reaction
PFS: Progression-free survival
RASSF1A: RAS association domain family 1, form A
RUNX3: Runt-related transcription factor 3
TP53: Tumor Protein 53
WES: Whole exome sequencing
WGS: Whole genome sequencing
